# Towards a Continuous Biometric System Based on ECG Signals Acquired on the Steering Wheel

**DOI:** 10.3390/s17102228

**Published:** 2017-09-28

**Authors:** João Ribeiro Pinto, Jaime S. Cardoso, André Lourenço, Carlos Carreiras

**Affiliations:** 1 Faculdade de Engenharia, Universidade do Porto; R. Dr. Roberto Frias, 4200-465 Porto, Portugal; jsc@fe.up.pt or jaime.cardoso@inesctec.pt; 2 INESC-TEC; R. Dr. Roberto Frias, 4200-465 Porto, Portugal; 3 CardioID Technologies Lda.; R. Adriano Correia de Oliveira 4A F1, 1600-312 Lisboa, Portugal; arl@cardio-id.com (A.L.); cac@cardio-id.com (C.C.); 4 Instituto Superior de Engenharia de Lisboa; R. Conselheiro Emídio Navarro 1, 1959-007 Lisboa, Portugal

**Keywords:** authentication, biometrics, continuous, electrocardiogram (ECG), identification, off-the-person, outlier detection, signal denoising

## Abstract

Electrocardiogram signals acquired through a steering wheel could be the key to seamless, highly comfortable, and continuous human recognition in driving settings. This paper focuses on the enhancement of the unprecedented lesser quality of such signals, through the combination of Savitzky-Golay and moving average filters, followed by outlier detection and removal based on normalised cross-correlation and clustering, which was able to render ensemble heartbeats of significantly higher quality. Discrete Cosine Transform (DCT) and Haar transform features were extracted and fed to decision methods based on Support Vector Machines (SVM), k-Nearest Neighbours (kNN), Multilayer Perceptrons (MLP), and Gaussian Mixture Models - Universal Background Models (GMM-UBM) classifiers, for both identification and authentication tasks. Additional techniques of user-tuned authentication and past score weighting were also studied. The method’s performance was comparable to some of the best recent state-of-the-art methods (94.9% identification rate (IDR) and 2.66% authentication equal error rate (EER)), despite lesser results with scarce train data (70.9% IDR and 11.8% EER). It was concluded that the method was suitable for biometric recognition with driving electrocardiogram signals, and could, with future developments, be used on a continuous system in seamless and highly noisy settings.

## 1. Introduction

Biometric recognition is gradually becoming a part of our daily lives, as it replaces common identification and access control systems based on keys, cards, codes, or passwords [1,2]. While these can be lost, copied, or stolen, biometric systems are based on intrinsic traits that are always with the person and ensure the correspondence between the subject’s and the credential’s identities [2,3].

The electrocardiogram (ECG), resulting from the electrical conduction through the heart needed for its contraction, is one of the most recent traits to be explored for biometric purposes [4,5]. Despite being far from as developed or widespread as face or fingerprint biometrics, the ECG offers unique advantages in terms of universality, uniqueness, permanence, and liveness assurance, that attest its potential for the recognition of individuals [5,6].

The use of the electrocardiogram as a biometric trait was first hypothesised in a 1977 US military report [7]. Despite the noticeably lesser acquisition quality, when compared with current measurement systems, ECG was nevertheless considered by this document as a very promising trait. Hoekema et al. [8] and van Oosterom et al. [9] strengthened this claim by studying in detail the intersubject variability of the ECG.

Biel et al. [10] and Kyoso et al. [11,12], from 1999 to 2001, were the first researchers working on this field. Biel et al. used features directly output by an ECG medical acquisition device, from Lead I, and performed decisions using Principal Component Analysis (PCA) and Soft Independent Modelling of Class Analogy (SIMCA), obtaining 100% identification rate (IDR) with 20 subjects. Kyoso et al. extracted fiducial latency features from CM5 lead signals, and attained 99.5% and 94.2% IDR (with three and nine subjects, respectively), using Mahalanobis distance and Linear Discriminant Analysis (LDA).

In opposition to medical signals, off-the-person signals are quickly becoming commonplace. This designation refers to ECG signals acquired on the fingers or palms of the subjects, using un-gelled electrodes, for higher acceptability and comfort during acquisition, despite the increasing need to overcome significant quality deterioration [13,14]. Chan et al. [15] were the first researchers to explore these acquisition settings for biometrics, with metallic electrodes at the thumbs, obtaining 89% IDR. Coutinho et al. [16] followed, by acquiring signals from the palms, using a conductive mat next to a computer keyboard, and reaching 99.5% IDR. More recently, an off-the-person collection was developed by a group of researchers from the University of Toronto [17] and was used by Louis et al. [18] for authentication, rendering a 7.89% Equal Error Rate (EER) with 1012 subjects.

ECG, being a continuously available signal, opens possibilities for the development of continuous or real-time recognition systems, which is especially advantageous for security or surveillance purposes. Guennoun et al. [19] were the first to explore such systems, for authentication, using fiducial features and Mahalanobis distance, and made decisions according to the individual matching of 35 consecutive heartbeats, obtaining 0.01% False Rejection Rate (FRR) and 0% False Acceptance Rate (FAR). Matta et al. [20] pioneered continuous identification, assessing identity every five seconds with 75% IDR, using autocorrelation features and LDA.

Other aspects that have been explored in ECG biometrics pertain to the effects of heart rate variability, different leads used, and long-term acquisitions. Pathoumvanh et al. [21] verified that the performance (IDR) of their system, based on continuous wavelet transform features and euclidean distance, decreased from 97% to 80% when using signals acquired after exercise. Ye et al. [22] observed that the performance, using Discrete Wavelet Transform (DWT) and Independent Component Analysis (ICA) features with Radial Basis Function (RBF) Support Vector Machines (SVM), on long-term signals is consistently worse than short-term.

Fang et al. [23] and Zhang et al. [24] have concluded, respectively, that using one lead renders significantly worse results than three leads, and that using limb leads such as I or II decreases the performance relative to the use of chest leads V1 or V2. This proves the additional difficulty placed upon off-the-person signals.

The state-of-the-art verifies a clear predominance of bandpass, highpass, lowpass, and/or notch filters for preprocessing of the signals [5,13,14,18,25,26,27,28,29,30,31,32,33,34], with fewer researchers opting for line fitting [35,36], DWT [37,38,39,40,41], or Discrete Cosine Transform (DCT) denoising [42]. Reference point detection has been performed in most research works [18,31,33,36,43] as well as signal segmentation in heartbeats [14,18,44] or fixed-size windows [20,30,45].

In what concerns features used, the most frequently extracted and successful were autocorrelation coefficients [5,25,39,46], Fourier, cosine, or wavelet transforms’ coefficients [22,26,28,30,34,47], Ensemble Empirical Mode Decomposition (EEMD) [48], averaged ensemble heartbeats [14,27,31,37,42], and several combinations of fiducial amplitude and time measurements [11,12,20,31,49,50]. Decision has been mainly performed through Nearest Neighbours or thresholding [5,14,28,32,35,36,51], SVM [6,22,27,39], or Artificial Neural Networks [40,50,52].

Despite all the evolutions the field has experienced throughout the last two decades, there is still much to overcome. The initiative described in this paper aims to take a leap forward in off-the-person ECG biometrics in a driving environment, useful for automatic personalisation of settings, supervision of professional drivers in fleets, and modelling of driving patterns for detection of distraction moments and fatigue-related accidents [53,54]. It achieved this by overcoming unprecedented noise and signal loss hurdles, characteristic of signals acquired whilst driving, using electrodes embedded seamlessly in a steering wheel cover. Through this, it also aimed to contribute towards reliable continuous ECG-based biometric recognition, using signals acquired in more comfortable and seamless configurations.

## 2. Proposed Methodology

**Figure 1:**
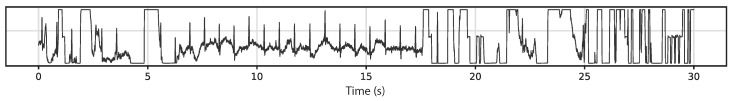
Example excerpt of the signal used, acquired on the steering wheel whilst driving. It is relevant to remark the evident and unprecedented predominance of noise over the signal, especially the effect of varying impedance denoted by the frequent saturation periods, which pose significant threats to the reliability of the recognition process.

The devised method aimed for the development of a biometric system that works using ECG signals to frequently recognise the driver. The signals used were acquired continuously and seamlessly on the steering wheel surface of a car. While, in state-of-the-art research, the contact of the subject with the sensor is guaranteed, the frequent hand movements required by the driving activity caused frequent contact loss and saturation periods. These give the signals unprecedented lesser quality, constituting a challenge for the recognition task (Figure 1).

After acquisition, the system ensured the relevance of the obtained signal by rejecting samples acquired when the hands were not on the wheel (confirmed by a hardware lead-on detector). Continuous contact periods were partitioned into segments of 5 s and overlap of 4 s, to allow for an initial decision after just five seconds of contact with the wheel, and a renewal of the decision each subsequent second.

**Figure 2:**
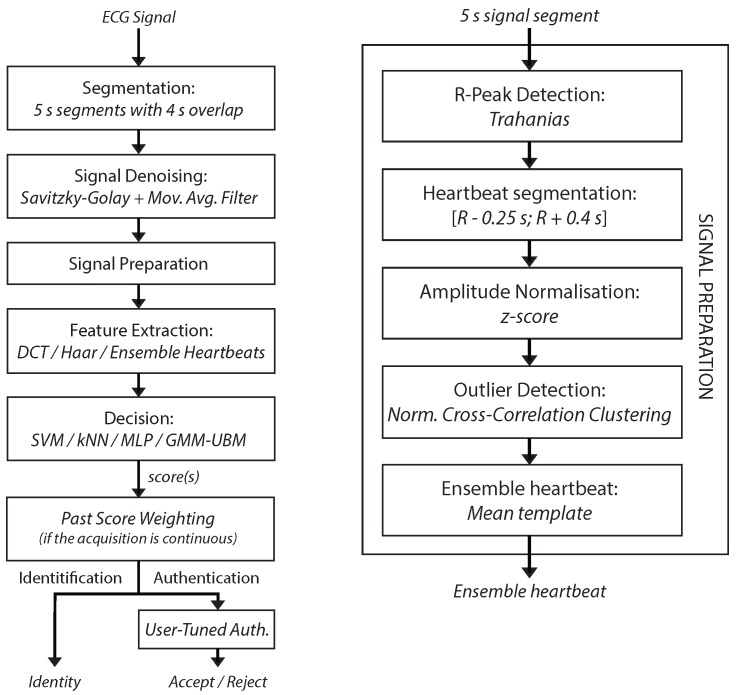
Overview of the proposed method, from acquisition to recognition (**left**), and the detailed process of the signal preparation block (**right**).

The process applied to each five-second frame (Figure 2) is presented in detail over the following subsections.

### 2.1. Signal Denoising

Common ECG signals used in prior art for biometrics come from either medical acquisitions or, more recently, off-the-person settings. Both of these contaminate the signal with noise, however, it is usually confined to 50/60 Hz (powerline interference) and 0–1 Hz (baseline wander from movement, breathing, and others) [55,56]. Noise in the acquired driving signals is noticeably more dominant and less predictable.

Thus, while methods like bandpass filters [14,18,25,27] or DCT denoising [42] have sufficed in the past, these more adverse signals require methods that make less assumptions on the noise nature and characteristics. The selected approach consisted of a combination of Savitzky-Golay with a moving average filter.

Savitzky-Golay [57] is based on least-squares error minimisation, by fitting a smooth polynomial line among the points in the neighbourhood of a sample and adjusting the latter’s amplitude to the fitted line’s. This method has excelled, in diverse past applications, in smoothing the signal by removing high frequency noise without causing distortion [35,58]. The moving average filter, based on an implementation through convolution using a 1 s window with overlap, served to complement the action of Savitzky-Golay, by removing low frequency noise, that the latter is unable to clean.

### 2.2. Signal Preparation

The process of denoising allowed for much cleaner signals. However, distortions like impedance variations and sensor saturation effects (caused by drivers grabbing the wheel with varying tightness), were not adequately removed by the previous phase.

Hence, the signal preparation step (Figure 2) served as intermediate process between denoising and feature extraction, aiming to reject saturation or unacceptably noisy signal segments, which could harm the recognition task.

#### 2.2.1. R-Peak Detection

The fiducial detection required for the segmentation of heartbeats was performed using the algorithm of Trahanias [59]. The algorithm is composed of a filtering phase, which computes the average between the signal after opening followed by closing and the signal after closing followed by opening. Then, a Peak-Valley Extraction (PVE) phase subtracts the signal after opening and closing the filtered signal. This combination of morphological operations enhances the sharpest variations on the signal, which are considered R-peak candidates. Among these, the R-peaks are selected through adaptive thresholding.

#### 2.2.2. Heartbeat Segmentation

The detected R-peaks were then used to obtain heartbeat segments. The segmentation was based on a fixed-window cropping of the signal, 0.25 s before each R-peak and 0.40 s after, as an adaptation of the successful approach proposed by Silva et al. [13]. For all heartbeats analysed during this work, this technique guaranteed the inclusion of all complete waveforms, regardless of heart rate or the duration changes it causes.

#### 2.2.3. Amplitude Normalisation

After segmentation, a normalised version *y* of each heartbeat segment *x* is obtained through *z-score* normalisation [30], by subtracting the segment’s mean amplitude (μ(x)), and dividing by the standard deviation (σ(x)):

(1) $$y\left[n\right]=\frac{x[n]-\mu (x)}{\sigma (x)}$$

This method provides greater similarity between cleaner heartbeats in all their extension, which benefited greatly the proposed method, especially in the step of the outlier detection. Time normalisation would also be beneficial, due to the varying heart rates while driving. However, it was discarded as it required the localisation of waveform onsets and offsets—highly unreliable in these noisy signals.

#### 2.2.4. Outlier Detection and Removal

For the task of identifying and rejecting outliers among the set of heartbeat candidates retrieved from the five-second segments, a novel approach was devised based on clustering and normalised cross-correlation.

Designated as NCCC (Normalised Cross-Correlation Clustering), the algorithm is centred on the assumption that clean heartbeats of a certain subject, in a short period of time, are very similar between themselves, while false or highly noisy heartbeats are random and different from both clean heartbeats and other false beats.

NCCC consists of the following steps:

1. Compute the normalised cross-correlation between each template ($x_{i}$) on the set and each of the others ($x_{j}$), with i,j=1,…,N. From all coefficients obtained, store in $NCC_{ij}$ only the maximum.
2. Get the average normalised cross-correlation for each template:
  (2) $$A_{i}=\frac{1}{N}\sum_{j=1}^{N}NCC_{ij};$$
3. Arrange *A* in descending order, and set an initial cluster with the *n* first templates;
4. Get the mean *m* of the cluster, and compute $\epsilon =\epsilon_{0}*m^{2}$;
5. Add the next template to the cluster if $m-A_{i}⩽\epsilon \wedge A_{i}⩾0.5$;
6. Repeat steps 4 and 5 until a template is rejected.

In this specific application, n=3 and $\epsilon_{0}=0.1$ were used. NCCC allowed the selection of only the templates most correlated with the others and, thus, least likely to be an outlier.

#### 2.2.5. Ensemble Construction

The process of signal preparation ends with the construction of an ensemble heartbeat, by averaging the templates selected by NCCC.

### 2.3. Feature Extraction

Feature extraction aims to capture the individual information present in each ensemble heartbeat, minimising the redundant information, to more efficiently discriminate between individuals. Towards this goal, fiducial and time domain features have been widely chosen on the prior art [29,45,50,60,61].

However, given the variability and noise present in the signals, frequency domain features were selected from the coefficients of DCT and Haar transforms.

- Discrete Cosine transform: The DCT coefficients were extracted from the ensemble heartbeats. The coefficients selected correspond to the frequency range [0, 40] Hz (total of 52 features);
- Haar Wavelet transform: The set of detail coefficients of the second level of decomposition with DWT using Haar wavelets was experimentally selected to serve as feature set for recognition (total of 163 features).

### 2.4. Recognition

Given a feature vector, recognition models compute and output scores for the claimed identity (for authentication tasks) or for each enrolled identity (for identification tasks). The models explored were:

- Support Vector Machines (SVM): SVM compute an optimal hyperplane dividing two classes, ensuring maximum margin between this boundary and the nearest samples. Kernels can be used to work with non-linearly separable datasets, and multiclass problems can be solved by combining binary classifiers [62];
- k-Nearest-Neighbours (kNN): kNN is a non-parametric, non-linear classifier. Based on the location of the object to be classified, kNN will find the *k* nearest train samples and predict the class most frequently verified [63];
- Multilayer Perceptrons (MLP): Multilayer Perceptrons are composed by neurons, which apply non-linear operations to their inputs. These are disposed in an input layer, which receives the features; an output layer, which outputs class scores; and a variable number of hidden layers in between. The connections between the neurons have their weights trained through error backpropagation [63];
- Gaussian Mixture Models - Universal Background Models (GMM-UBM): GMM models the distribution of the samples of each individual as a set of normal distributions, whose parameters can be used to classify unknown objects. UBM offers advantages in scarce training situations, by training the model with all samples first, and only then adapting for each subject [64].

Additionally, after the computation of scores by the decision methods mentioned above, in order to enhance the performance of the system by taking advantage of the availability of a claimed identity and the continuous nature of the data, two new techniques were proposed (Figure 2):

- User-tuned authentication: This technique was inspired by the notion of individuality among subjects exposed on Biometric Menagerie [65,66]. As subjects are unique, user-tuned authentication used a bespoke threshold/reference for acceptance/rejection for each enrolled individual, instead of a single threshold shared by all;
- Past score weighting: Outliers are expected to be frequent in highly noisy settings. Past Score weighting aims to reduce outlier influence by adjusting the most recent score using past scores, weighted by their recency. With $p_{i}\left(x_{t}\right)$ as the probability of the current sample belonging to class *i*, the weighted score was computed through:
  (3) $$p_{i}^{w}\left(x_{t}\right)=\frac{1}{\sum_{n=0}^{N}w_{t-n}}\sum_{n=0}^{N}w_{t-n}p_{i}\left(x_{t-n}\right)$$
  In Equation (4), *N* denotes the number of past scores to consider, and score weights $w_{t-n}$ were computed through a half-Gaussian function with tunable parameters:
  (4) $$w_{t-n}=e^{-\frac{{\left(t_{n}-t_{0}\right)}^{2}}{2\sigma^{2}}}$$

## 3. Results and Discussion

The proposed method was evaluated with excerpts of a continuous ECG recording, acquired over a period of approximately six days. Two excerpts, each with duration of 169–367 s, were randomly selected from two trips of the six drivers and combined in succession to simulate frequent and quick (15–25 s) driver swaps. The two trips of each driver were selected from different days, to maximise the time interval between the excerpts, with the exception of one subject, that only performed one trip.

The signal was acquired with a steering wheel cover, including periods of highway and city driving (higher activity), driver swaps, and idle periods. To the best of our knowledge, no ECG collection in such settings is yet available for use in biometrics research.

### 3.1. Signal Denoising

The proposed combination of Savitzky-Golay with a moving average filter (SG + MAF) had its performance compared with the most successful prior art methods: bandpass filters (BPF) with bands 1–40 Hz [18,25,26], 2–40 Hz [49,61], 1–30 Hz [27], and 2–30 Hz [16,29]; Savitzky-Golay (SG) [36]; Discrete Cosine Transform (DCT) [42]; and the combinations of Discrete Wavelet Transform with a moving average filter (DWT + MAF) [55] and with a highpass filter (DWT + HPF) [40].

**Figure 3:**
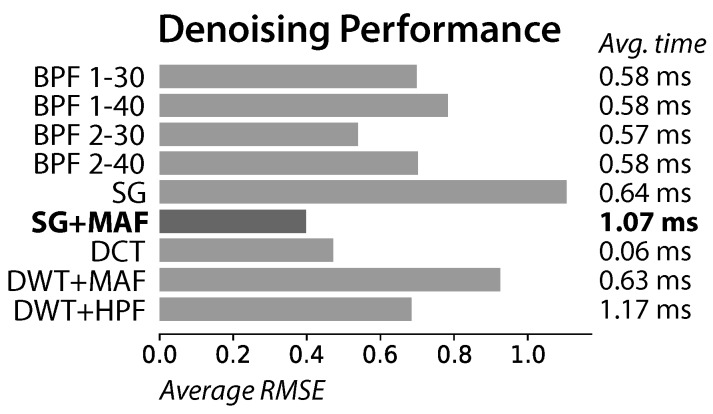
Root mean square error between the clean simulated signals and their versions after contamination with expected noise and denoising with each method. The times required to perform the denoising are also presented.

**Figure 4:**
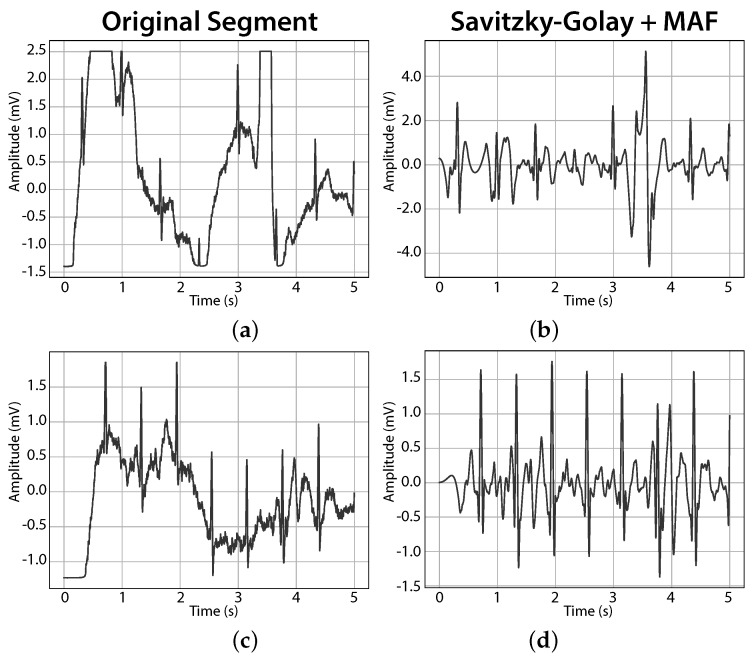
Denoising results in two example five-second segments (the signals were z-score normalised for visualisation; (**a**) first example segment; (**b**) first segment after denoising; (**c**) second example segment; (**d**) second segment after denoising). The proposed combination of Savitzky-Golay and moving average filter was able to adequately clean high frequency noise and baseline wander, despite the persistence of saturation effects.

The proposed method excelled with both simulated (clean simulated ECG signals using ECGSYN [67], contaminated with noise expected on driving settings, Figure 3) and real driving signals (Figure 4), by smoothing the signals and avoiding distortion in the best way.

### 3.2. Outlier Detection and Removal

**Figure 5:**
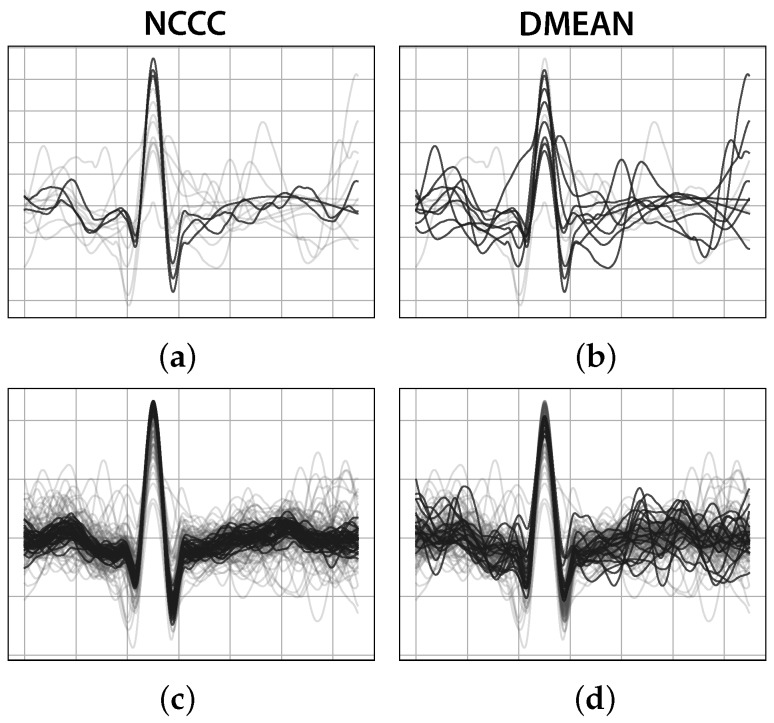
Comparison between DMEAN and NCCC, the proposed approach for outlier detection and removal, with two example template sets (first row: first template set with NCCC (**a**) and DMEAN (**b**); second row: second template set with NCCC (**c**) and DMEAN (**d**); dark lines: Selected heartbeats; light grey lines: Templates rejected as outliers).

The proposed NCCC method was compared with DMEAN [68] in a total of thirty template sets, extracted from segments 5 s to four minutes long, including six to 249 heartbeat templates each (Figure 5). The results were visually analysed, and the number of templates marked as outliers, the evolution of standard deviation before and after rejection, and the percentage of outliers with initial standard deviation between templates were recorded.

Despite DMEAN’s proven effectiveness with cleaner ECG signals, NCCC rejected more noisy and false heartbeats, and more effectively reduced the variance between templates in the set, while keeping most of the cleaner heartbeats. Nevertheless, it was consistently more computationally expensive than DMEAN.

### 3.3. Features and Recognition

**Figure 6:**
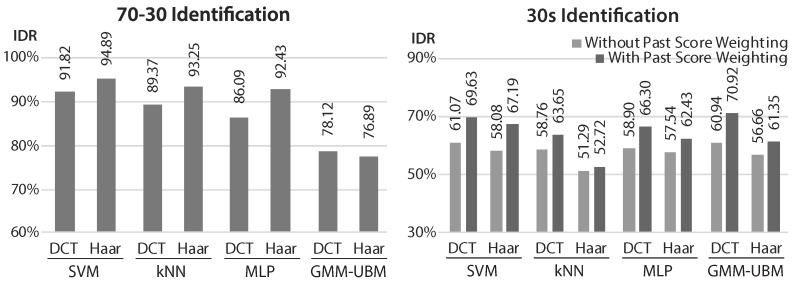
Identification rate (IDR, accuracy) results of the proposed method in identification tasks (left: Results with 70-30 dataset split; right: Results with 30 s train, with and without past score weighting).

**Figure 7:**
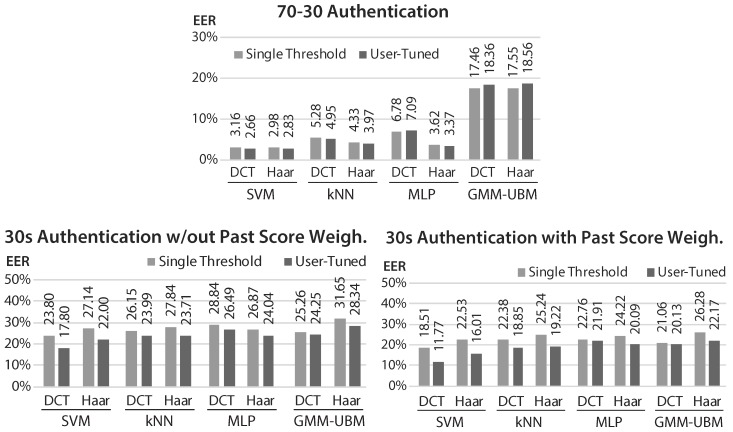
Equal error rate (EER) results of the proposed method in authentication tasks, with and without user-tuned thresholds (top: results with 70-30 dataset split; bottom-left: results with 30 s train; bottom-right: results with 30 s train and past score weighting).

The proposed approach was tested for identification and authentication tasks (results presented, respectively, in Figure 6 and Figure 7), in two settings: the first, using 70% of the data of each subject for train and 30% for testing, with cross-validation; and the second, using solely the first 30 s of data of each subject for train.

As stated in the proposed methodology, for DCT features, the coefficients that correspond to the frequency range [0, 40] Hz were chosen, as this narrow range contains all useful ECG information. As for Haar features, the detail coefficients of the various levels of decomposition were evaluated in cross-validation identification tasks, individually and combined. The set of coefficients of the second level was found to offer the best combination of better results and reduced dimensionality.

The first setting, 70-30, aimed to allow benchmarking with prior art methods. In both tasks, SVM was the best option, with results similar to some of the best state-of-the-art methods. There was no clear difference between using DCT or Haar features, neither in performance nor in extraction and decision times.

For the scarce data setting, the results were expectedly worse. In authentication, SVM with DCT features remained the best option, but GMM-UBM offered the best result for identification.

User-tuned authentication and Past Score Weighting brought, as shown, significant and consistent improvements in the performance results. It is expected that, with the integration of robust template/model update techniques, these results would experience further improvements.

## 4. Conclusions

The evident noise predominance over the ECG signals significantly decreased their quality. Nevertheless, the denoising with Savitzky-Golay and a moving average filter and the signal preparation process, that included the formulated Normalised Cross-Correlation Clustering outlier detection algorithm, allowed for the attainment of clean ensemble heartbeats.

These, after feature extraction with Discrete Cosine Transform, and decision with SVM, allowed for the overall best results in both identification and authentication. Moreover, the proposed techniques of User-Tuned Authentication and Past Score Weighting significantly enhanced the method’s performance.

Despite the need for further improvements, especially in continuous settings, this paper proves the feasibility of off-the-person ECG biometrics in driving settings. Although there are currently no signal collections available on these conditions, and benchmarking is thus unreliable, the method performed similarly to recent state-of-the-art approaches that used much cleaner signals, generally from medical acquisitions. Thus, the method proved able to recognise individuals using driving ECG signals, and it paved the way towards robust continuous ECG-based biometrics from seamless and highly noisy acquisition settings.

## Abbreviations

The following abbreviations are used in this manuscript:

BPF	Bandpass Filter
DCT	Discrete Cosine Transform
DWT	Discrete Wavelet Transform
ECG	Electrocardiogram
EEMD	Ensemble Empirical Mode Decomposition
EER	Equal Error Rate
FAR	False Acceptance Rate
FRR	False Rejection Rate
GMM	Gaussian Mixture Models
HPF	Highpass Filter
IDR	Identification Rate
kNN	k-Nearest Neighbours
LDA	Linear Discriminant Analysis
MAF	Moving Average Filter
MLP	Multilayer Perceptron
NCCC	Normalised Cross-Correlation Clustering
PCA	Principal Component Analysis
RBF	Radial Basis Function
SG	Savitzky-Golay
SIMCA	Soft Independent Modelling of Class Analogy
SVM	Support Vector Machines
UBM	Universal Background Models

## References

[1] Agrafioti F., Gao J., Hatzinakos D., Yang J. (2011). Heart Biometrics: Theory, Methods and Applications. Biometrics.

[2] Jain A.K., Ross A.A., Nandakumar K. (2011). Introduction to Biometrics.

[3] Kaur G., Singh G., Kumar V. (2014). A Review on Biometric Recognition. Int. J. Bio-Sci. Bio-Technol..

[4] Abo-Zahhad M., Ahmed S.M., Abbas S.N. (2014). Biometric authentication based on PCG and ECG signals: Present status and future directions. Signal Image Video Process..

[5] Agrafioti F., Bui F.M., Hatzinakos D. (2012). Secure Telemedicine: Biometrics for Remote and Continuous Patient Verification. J. Comput. Netw. Commun..

[6] Li M., Narayanan S. Robust ECG Biometrics by Fusing Temporal and Cepstral Information. Proceedings of the 2010 20th International Conference on Pattern Recognition (ICPR).

[7] Forsen G.E., Nelson M.R., Staron R.J. (1977). Personal Attributes Authentication Techniques.

[8] Hoekema R., Uijen G.J.H., van Oosterom A. (1999). Geometrical aspects of the inter-individual variability of multilead ECG recordings. Comput. Cardiol..

[9] Van Oosterom A., Hoekema R., Uijen G. (2000). Geometrical factors affecting the interindividual variability of the ECG and the VCG. J. Electrocardiol..

[10] Biel L., Pettersson O., Philipson L., Wide P. ECG analysis: A new approach in human identification. Proceedings of the 16th IEEE Instrumentation and Measurement Technology Conference (IMTC/99).

[11] Kyoso M., Ohishi K., Uchiyama A. (2000). Development of ECG Identification System. Jpn. J. Med. Electron. Biol. Eng..

[12] Kyoso M., Uchiyama A. Development of an ECG identification system. Proceedings of the 23rd Annual International Conference of the IEEE Engineering in Medicine and Biology Society.

[13] Silva H.P., Fred A., Lourenço A., Jain A.K. Finger ECG signal for user authentication: Usability and performance. Proceedings of the 2013 IEEE Sixth International Conference on Biometrics: Theory, Applications and Systems (BTAS).

[14] Carreiras C., Lourenço A., Silva H., Fred A., Ferreira R., Filipe J., Gusikhin O., Madani K., Sasiadek J. (2016). Evaluating Template Uniqueness in ECG Biometrics. Proceedings of the 11th International Conference on Informatics in Control, Automation and Robotics (ICINCO 2014), Vienna, Austria, 2–4 September 2014; Revised Selected Papers.

[15] Chan A.D.C., Hamdy M.M., Badre A., Badee V. (2008). Wavelet Distance Measure for Person Identification Using Electrocardiograms. IEEE Trans. Instrum. Meas..

[16] Coutinho D.P., Fred A.L.N., Figueiredo M.A.T. One-Lead ECG-Based Personal Identification Using Ziv-Merhav Cross Parsing. Proceedings of the 2010 20th International Conference on Pattern Recognition (ICPR).

[17] Wahabi S., Pouryayevali S., Hari S., Hatzinakos D. (2014). On Evaluating ECG Biometric Systems: Session-Dependence and Body Posture. IEEE Trans. Inf. Forensics Secur..

[18] Louis W., Komeili M., Hatzinakos D. (2016). Continuous Authentication Using One-Dimensional Multi-Resolution Local Binary Patterns (1DMRLBP) in ECG Biometrics. IEEE Trans. Inf. Forensics Secur..

[19] Guennoun M., Abbad N., Talom J., Rahman S.M.M., El-Khatib K. Continuous authentication by electrocardiogram data. Proceedings of the 2009 IEEE Toronto International Conference on Science and Technology for Humanity (TIC-STH).

[20] Matta R., Lau J.K.H., Agrafioti F., Hatzinakos D. Real-time continuous identification system using ECG signals. Proceedings of the 2011 24th Canadian Conference on Electrical and Computer Engineering (CCECE).

[21] Pathoumvanh S., Airphaiboon S., Hamamoto K. (2014). Robustness study of ECG biometric identification in heart rate variability conditions. IEEJ Trans. Electric. Electron. Eng..

[22] Ye C., Coimbra M.T., Kumar B.V.K.V. Investigation of human identification using two-lead Electrocardiogram (ECG) signals. Proceedings of the 2010 Fourth IEEE International Conference on Biometrics: Theory Applications and Systems (BTAS).

[23] Fang S.C., Chan H.L. (2009). Human identification by quantifying similarity and dissimilarity in electrocardiogram phase space. Pattern Recognit..

[24] Zhang Z., Wei D. A New ECG Identification Method Using Bayes’ Theorem. Proceedings of the TENCON 2006—2006 IEEE Region 10 Conference.

[25] Agrafioti F., Hatzinakos D. ECG Based Recognition Using Second Order Statistics. Proceedings of the 6th Annual Communication Networks and Services Research Conference (CNSR 2008).

[26] Belgacem N., Nait-Ali A., Fournier R., Bereksi-Reguig F. (2012). ECG based human authentication using wavelets and random forests. Int. J. Cryptogr. Inf. Secur..

[27] Lourenço A., Silva H., Fred A. ECG-based biometrics: A real time classification approach. Proceedings of the 2012 IEEE International Workshop on Machine Learning for Signal Processing.

[28] Matos A.C., Lourenço A., Nascimento J. Biometric recognition system using low bandwidth ECG signals. Proceedings of the 2013 IEEE 15th International Conference on e-Health Networking, Applications Services (Healthcom).

[29] Coutinho D.P., Silva H., Gamboa H., Fred A., Figueiredo M. (2013). Novel fiducial and non-fiducial approaches to electrocardiogram-based biometric systems. IET Biom..

[30] Odinaka I., Lai P.H., Kaplan A.D., O’Sullivan J.A., Sirevaag E.J., Kristjansson S.D., Sheffield A.K., Rohrbaugh J.W. ECG biometrics: A robust short-time frequency analysis. Proceedings of the 2010 IEEE International Workshop on Information Forensics and Security.

[31] Lourenço A., Silva H., Fred A. (2011). Unveiling the Biometric Potential of Finger-based ECG Signals. Intell. Neurosci..

[32] Porée F., Kervio G., Carrault G. (2016). ECG biometric analysis in different physiological recording conditions. Signal Image Video Process..

[33] Waili T., Nor R.M., Rahman A.W.B.A., Sidek K.A., Ibrahim A.A., Meesad P., Boonkrong S., Unger H. (2016). Electrocardiogram Identification: Use a Simple Set of Features in QRS Complex to Identify Individuals. Recent Advances in Information and Communication Technology 2016: Proceedings of the 12th International Conference on Computing and Information Technology (IC2IT), Khon Kaen, Thailand, 7–8 July 2016.

[34] Tan R., Perkowski M. (2017). Toward Improving Electrocardiogram (ECG) Biometric Verification using Mobile Sensors: A Two-Stage Classifier Approach. Sensors.

[35] Dar M.N., Akram M.U., Shaukat A., Khan M.A. ECG Based Biometric Identification for Population with Normal and Cardiac Anomalies Using Hybrid HRV and DWT Features. Proceedings of the 2015 5th International Conference on IT Convergence and Security (ICITCS).

[36] Molina G.G., Bruekers F., Presura C., Damstra M., van der Veen M. Morphological synthesis of ECG signals for person authentication. Proceedings of the 2007 15th European Signal Processing Conference.

[37] Fatemian S.Z., Agrafioti F., Hatzinakos D. HeartID: Cardiac biometric recognition. Proceedings of the 2010 Fourth IEEE International Conference on Biometrics: Theory, Applications and Systems (BTAS).

[38] Chun S.Y. Single pulse ECG-based small scale user authentication using guided filtering. Proceedings of the 2016 International Conference on Biometrics (ICB).

[39] Hejazi M., Al-Haddad S., Singh Y.P., Hashim S.J., Aziz A.F.A. (2016). ECG biometric authentication based on non-fiducial approach using kernel methods. Digit. Signal Process..

[40] Boumbarov O., Velchev Y., Sokolov S. ECG personal identification in subspaces using radial basis neural networks. Proceedings of the IEEE International Workshop on Intelligent Data Acquisition and Advanced Computing Systems: Technology and Applications (IDAACS 2009).

[41] Sasikala P., Wahidabanu R. (2010). Identification of Individuals using Electrocardiogram. Int. J. Comput. Sci. Netw. Secur..

[42] Choudhary T., Manikandan M.S. A novel unified framework for noise-robust ECG-based biometric authentication. Proceedings of the 2015 2nd International Conference on Signal Processing and Integrated Networks (SPIN).

[43] Venkatesh N., Jayaraman S. Human Electrocardiogram for Biometrics Using DTW and FLDA. Proceedings of the 2010 20th International Conference on Pattern Recognition (ICPR).

[44] Zhou X., Lu Y., Chen M., Bao S.D., Miao F. A method of ECG template extraction for biometrics applications. Proceedings of the 2014 36th Annual International Conference of the IEEE Engineering in Medicine and Biology Society.

[45] Ergin S., Uysal A.K., Gunal E.S., Gunal S., Gulmezoglu M.B. ECG based biometric authentication using ensemble of features. Proceedinsgs of the 2014 9th Iberian Conference on Information Systems and Technologies (CISTI).

[46] Plataniotis K.N., Hatzinakos D., Lee J.K.M. ECG Biometric Recognition Without Fiducial Detection. Proceedings of the 2006 Biometrics Symposium: Special Session on Research at the Biometric Consortium Conference.

[47] Saechia S., Koseeyaporn J., Wardkein P. Human Identification System Based ECG Signal. Proceedings of the TENCON 2005—2005 IEEE Region 10 Conference.

[48] Zhao Z., Yang L., Chen D., Luo Y. (2013). A Human ECG Identification System Based on Ensemble Empirical Mode Decomposition. Sensors.

[49] Israel S.A., Irvine J.M., Cheng A., Wiederhold M.D., Wiederhold B.K. (2005). ECG to identify individuals. Pattern Recognit..

[50] Waili T., Nor R.M., Yaacob H., Sidek K., Rahman A.W.A. A Hasty Approach to ECG Person Identification. Proceedings of the 2016 International Conference on Computer and Communication Engineering (ICCCE).

[51] Brás S., Pinho A.J. ECG biometric identification: A compression based approach. Proceedings of the 2015 37th Annual International Conference of the IEEE Engineering in Medicine and Biology Society (EMBC).

[52] Iqbal F.T.Z., Sidek K.A., Noah N.A., Gunawan T.S. A comparative analysis of QRS and cardioid graph based ECG biometric recognition in different physiological conditions. Proceedings of the IEEE International Conference on Smart Instrumentation, Measurement and Applications (ICSIMA2014).

[53] Lourenço A., Alves A.P., Carreiras C., Duarte R.P., Fred A., Bifet A., May M., Zadrozny B., Gavalda R., Pedreschi D., Bonchi F., Cardoso J., Spiliopoulou M. (2015). CardioWheel: ECG Biometrics on the Steering Wheel. Proceedings of the European Conference: Machine Learning and Knowledge Discovery in Databases (ECML PKDD 2015), Porto, Portugal, 7–11 September 2015.

[54] Hansen J.H.L., Busso C., Zheng Y., Sathyanarayana A. (2017). Driver Modeling for Detection and Assessment of Driver Distraction: Examples from the UTDrive Test Bed. IEEE Signal Process. Mag..

[55] Fatemian S.Z., Hatzinakos D. A new ECG feature extractor for biometric recognition. Proceedings of the 2009 16th International Conference on Digital Signal Processing.

[56] Singh B., Singh P., Budhiraja S. Various Approaches to Minimise Noises in ECG Signal: A Survey. Proceedings of the 2015 Fifth International Conference on Advanced Computing Communication Technologies.

[57] Savitzky A., Golay M. (1964). Smoothing and differentiation of data by simplified least squares procedures. Anal. Chem..

[58] Schafer R.W. (2011). What Is a Savitzky-Golay Filter? [Lecture Notes]. IEEE Signal Process. Mag..

[59] Trahanias P.E. (1993). An approach to QRS complex detection using mathematical morphology. IEEE Trans. Biomed. Eng..

[60] Shen T.W.D., Tompkins W.J., Hu Y.H. (2011). Implementation of a one-lead ECG human identification system on a normal population. J. Eng. Comput. Innov..

[61] Rezgui D., Lachiri Z. (2016). ECG biometric recognition using SVM-based approach. IEEJ Trans. Electric. Electron. Eng..

[62] Cortes C., Vapnik V. (1995). Support-vector networks. Mach. Learn..

[63] Theodoridis S., Koutroumbas K. (2009). Pattern Recognition.

[64] Reynolds D.A., Quatieri T.F., Dunn R.B. (2000). Speaker Verification Using Adapted Gaussian Mixture Models. Digit. Signal Process..

[65] Doddington G., Liggett W., Martin A., Przybocki M., Reynolds D. (1998). Sheep, Goats, Lambs and Wolves: A Statistical Analysis of Speaker Performance in the NIST 1998 Speaker Recognition Evaluation.

[66] Yager N., Dunstone T. (2010). The Biometric Menagerie. IEEE Trans. Pattern Anal. Mach. Intell..

[67] McSharry P.E., Clifford G.D., Tarassenko L., Smith L.A. (2003). A dynamical model for generating synthetic electrocardiogram signals. IEEE Trans. Biomed. Eng..

[68] Lourenço A., Silva H., Carreiras C., Fred A., Kamel M., Campilho A. (2013). Outlier Detection in Non-intrusive ECG Biometric System. Proceedings of the 10th International Conference on Image Analysis and Recognition (ICIAR 2013), Póvoa do Varzim, Portugal, 26–28 June 2013.

